# Mitochondrial ATP Synthase *beta*-Subunit Affects Plastid Retrograde Signaling in *Arabidopsis*

**DOI:** 10.3390/ijms25147829

**Published:** 2024-07-17

**Authors:** Hao Liu, Zhixin Liu, Aizhi Qin, Yaping Zhou, Susu Sun, Yumeng Liu, Mengke Hu, Jincheng Yang, Xuwu Sun

**Affiliations:** State Key Laboratory of Crop Stress Adaptation and Improvement, State Key Laboratory of Cotton Biology, Key Laboratory of Plant Stress Biology, School of Life Sciences, Henan University, 85 Minglun Street, Kaifeng 475001, China; lhao0520@126.com (H.L.); lzx2021@henu.edu.cn (Z.L.); qaz6835@henu.edu.cn (A.Q.); zhouyapinghenu@sina.com (Y.Z.); su17861523158@sina.com (S.S.); 13569882910@sina.com (Y.L.); mengkehu218511@sina.com (M.H.); 13458345215@sina.cn (J.Y.)

**Keywords:** *AT5G08670*, mitochondrial protein, ATP synthase *beta*-subunit, ATP synthase activity, plastid retrograde signaling

## Abstract

Plastid retrograde signaling plays a key role in coordinating the expression of plastid genes and photosynthesis-associated nuclear genes (PhANGs). Although plastid retrograde signaling can be substantially compromised by mitochondrial dysfunction, it is not yet clear whether specific mitochondrial factors are required to regulate plastid retrograde signaling. Here, we show that mitochondrial ATP synthase *beta*-subunit mutants with decreased ATP synthase activity are impaired in plastid retrograde signaling in *Arabidopsis thaliana*. Transcriptome analysis revealed that the expression levels of PhANGs were significantly higher in the mutants affected in the *AT5G08670* gene encoding the mitochondrial ATP synthase *beta*-subunit, compared to wild-type (WT) seedlings when treated with lincomycin (LIN) or norflurazon (NF). Further studies indicated that the expression of nuclear genes involved in chloroplast and mitochondrial retrograde signaling was affected in the *AT5G08670* mutant seedlings treated with LIN. These changes might be linked to the modulation of some transcription factors (TFs), such as *LHY* (*Late Elongated Hypocotyl*), *PIF* (*Phytochrome-Interacting Factors*), *MYB*, *WRKY*, and *AP2/ERF* (Ethylene Responsive Factors). These findings suggest that the activity of mitochondrial ATP synthase significantly influences plastid retrograde signaling.

## 1. Introduction

The chloroplast is a complex cellular organelle that not only performs photosynthesis but also synthesizes various macromolecules and metabolites including fatty acids, vitamins, tetrapyrroles, and amino acids required for plant growth [1,2]. Chloroplasts, as semi-autonomous organelles, are composed of proteins encoded by the nuclear and chloroplast genomes. The chloroplast genome encodes less than 100 proteins, whereas *Arabidopsis thaliana* has approximately 2100 photosynthesis-associated nuclear genes (PhANGs) encoding chloroplast proteins [3]. However, many essential components of the photosynthetic machinery are encoded by the chloroplast genome [4]. Therefore, the maintenance of homeostasis in the plastid requires not only regulation by the plastid itself but also coordination between the chloroplast and the nucleus through retrograde signals. Although the nucleus regulates the development and function of organelles, these organelles also transmit signals to the nucleus to deliver information regarding their growth and developmental status for adjusting the expression of nuclear genes [5]. For example, *CAB over-expression 1* (*COE1*) and *CAB over-expression 2* (*COE2*), involved in plastid retrograde signaling, significantly influence chloroplast biogenesis and plant growth [6,7]. Treatment of *Arabidopsis* seedlings with the plastid translation inhibitor lincomycin (LIN) or the carotenoid biosynthesis inhibitor norflurazon (NF) inhibits chloroplast biogenesis, promoting the chloroplast to send retrograde signals to the nucleus, down-regulating the expression of certain PhANGs. A genetic screen identified the *genomes-uncoupled 1* (*gun1*) mutant, which accumulated higher levels of endogenous *chlorophyll a/b-binding protein* (*CAB*) mRNA than in wild type when treated with NF [8]. Further mutant screening identified five additional *Arabidopsis* nuclear *gun* mutants, *gun2*~*gun6*, involved in the tetrapyrrole biosynthesis pathway (TBP), and these mutants failed to effectively repress the transcription of specific nuclear genes after NF treatment [8,9,10,11,12,13].

The intricate interplay between chloroplasts and mitochondria necessitates a synergistic coordination between photosynthesis and respiration [14]. Mitochondria and chloroplasts, the two energy-converting organelles of plants, are tightly coordinated to provide energy for the biological processes of plant cells [15]. In chloroplasts, photosynthesis uses light energy, carbon dioxide, and water to produce carbohydrates and evolve oxygen [16]. In mitochondria, respiration consumes organic matter and oxygen and results in the formation of ATP and oxidizing power with the release of carbon dioxide and water [17]. Therefore, the products of respiration and photosynthesis circulate between each other to achieve a balanced energy status. In addition, mitochondrial metabolism, especially oxidative electron transport, and phosphorylation, is crucial for photosynthetic carbon assimilation [18,19]. Mitochondria, through the *cytochrome c oxidase* (*COX*) and *alternative oxidase* (*AOX*) pathways, oxidize excess reducing equivalents from chloroplasts and thereby protect chloroplasts from photoinhibition [20,21]. The chloroplast supplies heme precursors to the mitochondria and metabolites that are involved in photorespiration, such as serine and glycine [22,23]. In summary, the functional interplay between mitochondria and chloroplasts likely modulates each organelle’s developmental and functional states, thereby influencing their respective retrograde signals.

In plants, the chloroplast-mitochondrial cross-talk is essential for various biological processes and for maintaining cell homeostasis. Genetic studies showed that the cross-talk between chloroplasts and mitochondria regulates organelle development. First, mitochondrial dysfunction can cause abnormalities in chloroplast development. Although the precise processes underlying the connections between respiration and photosynthesis remain unclear, it is intriguing to observe that all of the maize *nonchromosomal stripe* (*NCS*) mitochondrial mutants exhibit a severe photosynthetic defect [24]. Similarly, deficiencies of mitochondrial glycine decarboxylase complex in barley lead to over-reduction and over-energization of the chloroplast [25]. Mutations affecting the activities of mitochondrial glycine decarboxylase and nicotinamide adenine dinucleotide (NADH) dehydrogenase resulted in decreased photosynthesis and impaired photorespiration [25,26]. Reduced levels of P-protein, one of the components of mitochondrial glycine decarboxylase, inhibited the growth and photosynthetic activity of potatoes [27]. White striped leaves and white panicles are produced by a mutation in rice affecting the nuclear gene *WHITE PANICLE 3* encoding a mitochondrial protein. Significant developmental abnormalities do occur in mitochondria and chloroplasts in this mutant [28].

Numerous studies demonstrate a cross-talk between mitochondrial and chloroplast retrograde signals. The chloroplast represses PhANGs through plastid gene expression (PGE) signals and Mg–protoporphyrin IX (Mg–proto) [29,30]. Moreover, the PGE pathway and signals originating from impaired mitochondrial gene expression act together to repress PhANGs [23]. Activation of retrograde signals by 3′-phosphoadenosine 5′-phosphate (PAP) and 5′-3′ exoribonucleases (XRNs) influences nuclear gene expression through RNA processing or mRNA decay which leads to the accumulation of *phosphatase-like protein* (*SAL1*) in both mitochondria and chloroplasts and controls nuclear gene expression [15,31,32]. It is an essential component of mitochondrial retrograde signaling which regulates the expression of *AOX1a* and *Light-harvesting complex II chlorophyll a/b-binding protein 2.4* (*Lhcb2.4*) when the photosynthetic electron transport chain is impaired [33].

While several studies have revealed that mitochondrial defects affect chloroplast function and development, it is not yet clear whether decreased mitochondrial respiratory activity also affects the retrograde signaling of chloroplasts. To investigate this hypothesis, we chose T-DNA insertion mutants partially deficient in mitochondrial ATP synthase activity. Here, we demonstrate through various methodologies and transcriptome analysis that a reduction in mitochondrial ATP synthase activity affects plastid retrograde signaling.

## 2. Results

### 2.1. Isolation, Sequence Analysis, and Expression Pattern of Mitochondrial ATP Synthase beta-Subunit

**Figure 1:**
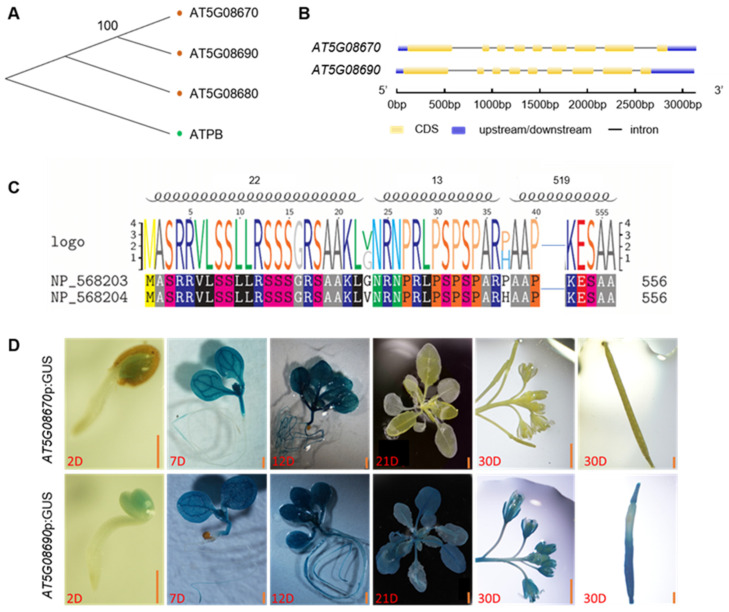
Sequence analysis and tissue-specific localization of mitochondrial ATP synthase *beta*-subunit. (**A**) Phylogenetic analysis of *beta*-subunits of mitochondrial and chloroplast ATP synthase was conducted with MEGA 7 using maximum-likelihood phylogenetic analysis with 1000 bootstrap replicates. (**B**) Gene structure diagram of *AT5G08670* and *AT5G08690*. (**C**) Alignment of amino acid sequences (NP_5683203 corresponds to *AT5G08670*, NP_5683204 corresponds to *AT5G08690*, and Helix corresponds to conserved domains). (**D**) GUS activity in *AT5G08670*p:GUS and *AT5G08690*p:GUS transgenic plants at different developmental stages. Scale bar, 1 mm.

The mitochondrial ATP synthase *beta*-subunit (ATPB) is encoded by three genes (*AT5G08670*, *AT5G08680*, *AT5G08690*) with highly similar amino acid sequences (98% sequence identity). The mature protein consists of 556 amino acids and has a molecular mass of 59,630 Da and an isoelectric point of 6.53. Multiple sequence alignment analysis and the phylogenetic tree show that mitochondrial and chloroplast *ATPB* genes cluster in two branches (Figure 1A). *AT5G08670* and *AT5G08690* are closer on the physiological tree (Figure 1A). They are spaced approximately 4400 bp apart, and their precursor proteins consist of 566 amino acids (Figure 1B,C). The localization of these proteins was determined using online protein-peptide programs (TARGET P 2.0) which predicted a mitochondrial presequence with a cleavage site localized between amino acids 51 and 52 (Figure S1). A comparison of the precursor proteins for *AT5G08690* (NP 568204) and *AT5G08670* (NP 568203) revealed that there are only two residues that differ between the two proteins (Figure 1C) within the presequence. To analyze the expression patterns of *AT5G08670* and *AT5G08690*, we generated transgenic plants expressing *AT5G08670*p:GUS, and *AT5G08690*p:GUS. These two lines showed a similar spatiotemporal expression pattern of *AT5G08670* and *AT5G08690* at the early developmental stages of seedlings. However, at later developmental stages, *AT5G08670* expression declined, and after 21 days, it was no longer expressed (Figure 1D). These findings indicate that these two proteins are highly similar although their spatiotemporal expression patterns are different, which might contribute to functional differences at subsequent developmental stages. GUS staining of *AT5G08670*p:GUS transgenic plants revealed *AT5G08670* is predominantly expressed in seedlings leaves, indicating its critical role in the early seedling development. Considering the differential expression of these two genes during the developmental stage, in this study, we primarily focused on the characterization of *AT5G08670*.

### 2.2. Subcellular Localization of the AT5G08670 Protein in Arabidopsis thaliana

**Figure 2:**
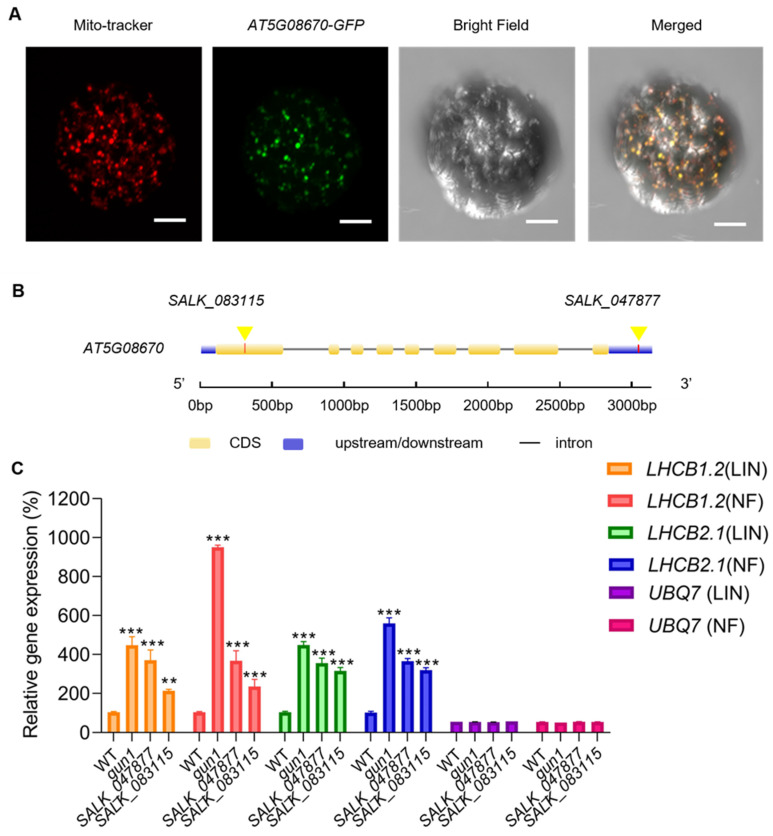
Subcellular localization of *AT5G08670* and gene expression level. (**A**) (1) Mito-Tracker red was used to show the distribution of mitochondria. (2) Distribution pattern of *AT5G08670-GFP* fusion protein. (3) Image of *Arabidopsis* protoplasts in a bright field. (4) Merged image of Mito-Tracker red, and *AT5G08670-GFP*, bright field. Scale bar, 10 µm. (**B**) The T-DNA insertion lines, *SALK_04787*7 and *SALK_083115* contain insertions within the 3′ UTR and exon 1 of the *AT5G08670*, respectively. Transcription proceeds from left to right. (**C**) The relative expression level of LHCB after LIN and NF treatment. The relative expression level of *LHCB1.2* and *LHCB2.1* in *gun1*, *SALK*_047877, *SALK_083115*, and WT plants after LIN and NF treatment. *UBQ7* was used as a reference gene, not responsive to LIN and NF. Significant differences are indicated by asterisks (one–way ANOVA with Tukey’s multiple comparisons test, ** *p* < 0.01, *** *p* < 0.001, *n* = 3).

In plant cells, ATP synthases are present in chloroplasts and mitochondria. The mitochondrial ATP synthase, also known as F1Fo-ATP synthase, catalyzes oxidative phosphorylation and uses the transmembrane proton gradient to synthesize ATP [34]. In public databases (The Arabidopsis Information Resource, TAIR; www.arabidopsis.org, accessed on 17 July 2020), *AT5G08670* is also annotated as a mitochondrial protein, encoding a mitochondrial ATP synthase *beta*-subunit. We used Mito-Tracker Red CMXRos, a red-fluorescent dye that stains mitochondria in cells and its accumulation within mitochondria depends on the mitochondrial membrane potential (Figure 2A). Merging the fluorescence image of protoplasts of *Arabidopsis* transiently expressing the *AT5G08670-GFP* fusion protein with the Mito-tracker image confirmed that *AT5G08670-GFP* is localized in mitochondria.

### 2.3. Loss of Function of AT5G08670 Leads to a Decrease in Mitochondrial ATP Synthase Level and Activity and Affects Plastid Retrograde Signaling

To determine the main functions of *AT5G08670*, T-DNA insertion mutants of *AT5G08670* (*SALK_047877* and *SALK_083115*) were obtained (Figure 2B), and the homozygous T-DNA insertion was confirmed (Figure S2A,B). Phenotypic analysis of two-week-old *SALK_047877* and *SALK_083115* revealed a marginally reduced size compared to wild-type plants (Figure S2C). To test whether *AT5G08670* is involved in plastid and mitochondrial retrograde signaling, we used the plastid development inhibitors norflurazon (NF) and lincomycin (LIN) which allow one to monitor plastid retrograde signaling. The *SALK_047877* and *SALK_083115* lines and *genomes uncoupled 1* (*gun1*) (as a positive control) were treated with LIN (220 µg/mL) and NF. Expression of *Light-Harvesting Chl a/b-Binding* (*LHCB*) genes (*LHCB1.2* and *LHCB2.1*) was determined by Quantitative Real-Time PCR (RT-qPCR) to be significantly higher in *gun1*, *SALK_047877*, and *SALK_083115* than in WT seedlings treated with LIN or NF (Figure 2C). To verify that the *SALK_047877* mutant phenotype is caused by a mutation in the *AT5G08670* gene, we performed a genetic complementation experiment using full-length CDS. A significantly increased expression level of *AT5G08670* could be detected in the complementary strain by Western blot, and the plant size of the complementary strain was more similar to that of WT (Figure S2C,D). These results suggest that *AT5G08670* may play a role in plastid retrograde signaling.

**Figure 3:**
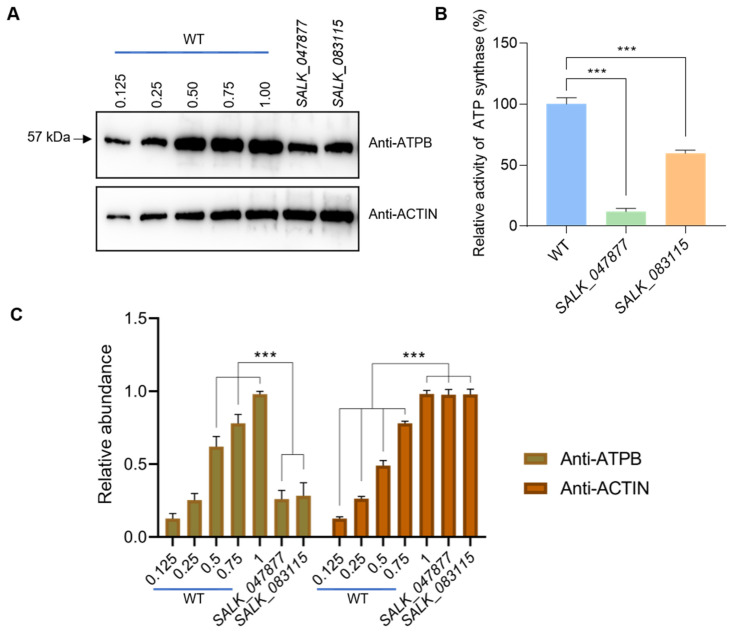
ATP synthase *beta*-subunit and total ATP synthase activity are decreased in the *AT5G08670* mutants. (**A**) Immunoblotting with anti-ATPB of mitochondrial ATP synthase *beta*-subunit in WT and *AT5G08670* mutants (*SALK_047877* and *SALK_083115*). In order to compare the relative levels of ATPB protein in *SALK_047877* and *SALK_083115*, we designed 5 loading gradients for WT protein, including 0.125, 0.25, 0.50, 0.75, and 1.00. The loading amount of samples *SALK_047877* and *SALK_083115* is equivalent to the level of 1.00 in WT. Anti-ACTIN is used as a loading control. (**B**) The activity of ATP synthase in WT and *AT5G08670* mutants (*SALK_047877* and *SALK_083115*). (**C**) Quantification of protein band of WT and mutants (*SALK_047877* and *SALK_083115*) by grayscale analysis. Significant differences are indicated by asterisks between WT and mutants (*SALK_047877* and *SALK_083115*). (One–way ANOVA with Tukey’s multiple comparisons test, *** *p* < 0.001, *n* = 3).

Given that *AT5G08670* and *AT5G08690* encode an identical mature mitochondrial ATP synthase *beta*-subunit, we investigated whether the levels and activity of ATP synthase were reduced in *AT5G08670* mutants. Immunoblotting with ATPB antibody revealed that the level of ATP synthase *beta*-subunit dropped to 25% of WT level in the *AT5G08670* mutants, and total ATP synthase activity also. The grayscale analysis of protein bands by Image J revealed that ATP synthase *beta*-subunit levels in *AT5G08670* mutants were significantly lower compared to those in WT (Figure 3A–C). Taken together, these results indicate that a decrease in mitochondrial ATP synthase activity affects retrograde signaling.

### 2.4. Detection of Differentially Expressed Genes (DEGs) in AT5G08670 Mutants by RNA-Sequencing (RNA-Seq)

**Figure 4:**
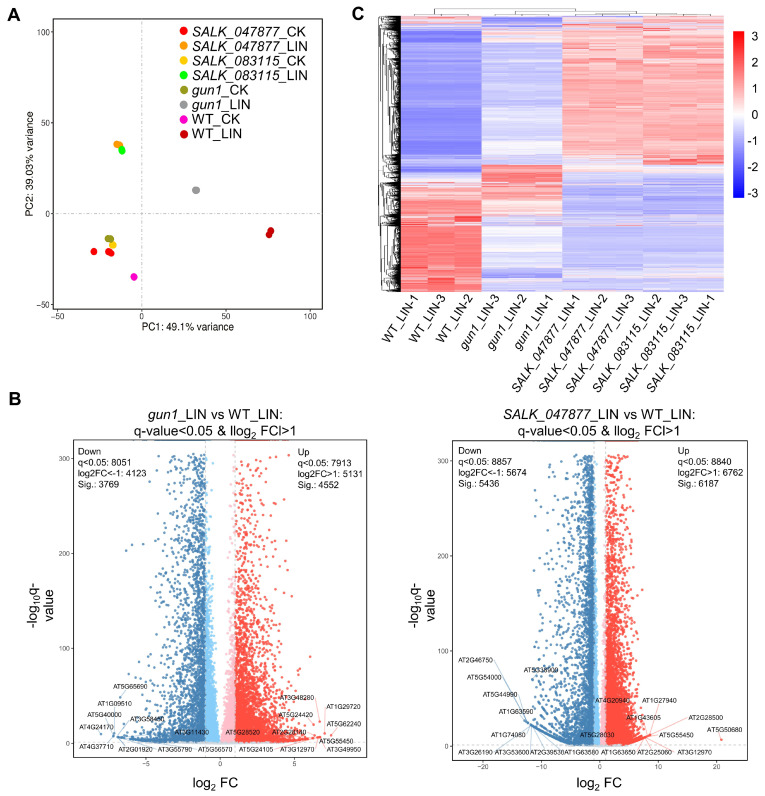
Analysis of DEGs after LIN treatment between mutants and WT. (**A**) Principal component analysis of gene expression in the experimental and control groups. The expression of the protein-coding genes was estimated in FPKM (Fragments Per kb Per Million Reads). PCA shows the relationship between samples from different dimensions. The closer the sample clustering distance or PCA distance is, the more similar the samples are. (**B**) Volcano plot of DEGs in *gun1*_LIN vs. WT_LIN, volcano plot of DEGs in *SALK_047877*_LIN vs. WT_LIN. (**C**) Heatmap showing the expression patterns of DEGs between the control group and LIN-treated group for WT and mutants by unsupervised hierarchical clustering of DEGs with LIN treatment (*p*-value < 0.05 and |log2FC| > 1).

RNA-seq analysis was performed to study the effects of the T-DNA insertions in *AT5G08670* on the transcriptome. RNA samples from 7-day-old WT, *SALK_047877*, *SALK_083115*, and *gun1* seedlings grown either under normal conditions or treated with LIN (220 µg/mL) were used for Illumina Genome Analyzer deep sequencing, a widely adopted next-generation sequencing (NGS) technology. Transcriptome sequencing analysis of 24 samples yielded 178.32 GB of clean data; among the high-quality clean reads, the percentage of the Q30 base was >93%, and the GC contents were >45%. These reference data indicated that the sequencing results are reliable and could be used for further analysis (Table S1). Approximately 97.33% of the reads were mapped to the reference genome of *Arabidopsis*; 50,291,382 reads were mapped to unique regions, and 1,034,888 were assigned to multiple areas (Table S2). The average mapping rate, which reached 97.33%, shows the high quality of the sequencing results. Principal component analysis (PCA) was performed using the expression of genes to examine the distribution of samples and explore relationships between samples. Samples in the same group were more concentrated in spatial distribution. Significant differences were found between the LIN-treated and control samples (Figure 4A). We generated three DEG comparison groups (volcano plot) in *gun1*_LIN vs. WT_LIN and *SALK_047877*_LIN vs. WT_LIN based on the criteria of a >2-fold change in expression and a significance test (*p*-value < 0.05), among which 4552 up-regulated and 3769 down-regulated genes were found in the *gun1*_LIN vs. WT_LIN comparison group (Figure 4B, Table S3). In the *SALK_047877*_LIN vs. WT_LIN comparison group, there were 6187 up-regulated and 5436 down-regulated genes (Figure 4B, Table S4). Heatmaps were used to show gene expression differences in WT, *gun1*, *SALK_047877*, and *SALK_083115* after LIN treatment (Figure 4C, Tables S3–S5). The expression patterns of the three samples of each component are similar, which proves that the repeatability of each sample is high, and the data obtained are also credible. Cluster analysis of each component showed that after LIN treatment *gun1* and SALK_lines clustered separately from WT, indicating that their overall gene expression pattern is different from that of WT.

### 2.5. Gene Ontology (GO) Enrichment Analysis of DEGs between WT and SALK Lines after LIN Treatment

**Figure 5:**
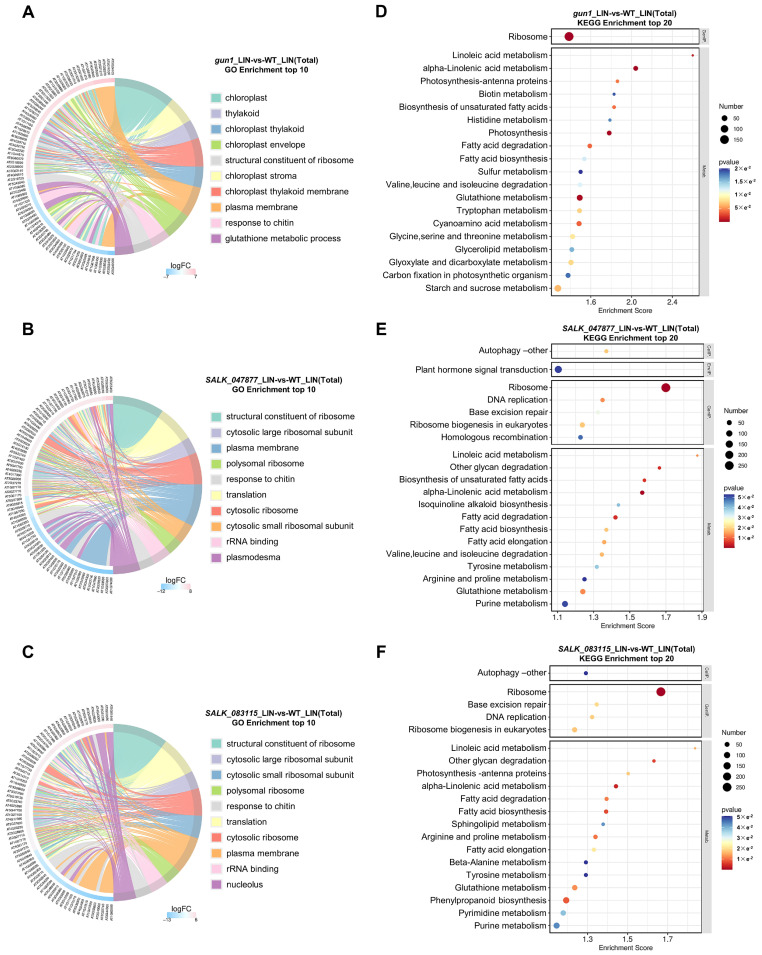
GO and KEGG enrichment analysis after LIN treatment. (**A**–**C**) GO enrichment analysis of DEGs, identified by comparison between WT, *gun1*, *SALK_047877*, and *SALK-083115* respectively under LIN treatment, in *gun1* (**A**), *SALK_047877* (**B**), and *SALK-083115* (**C**). (**D**–**F**) KEGG enrichment analysis of DEGs after LIN treatment in *gun1* (**D**), *SALK_047877* (**E**), and *SALK-083115* respectively (**F**).

A GO enrichment analysis of DEGs was carried out, and the top 10 GO terms with the highest FC DEGs were selected from each GO category for display. The GO enrichment analysis and visualization were performed using OECloud tools (https://cloud.oebiotech.cn, accessed on 5 March 2023). The GO analysis showed that the DEGs could be grouped based on their cellular location, functions, and biological processes in which they were involved. In *gun1*, the cellular component analysis showed that the most enriched portion of DEGs was involved in the chloroplast, followed by the thylakoid, chloroplast membrane, chloroplast stroma, and plasma membrane (Figure 5A). In terms of molecular functions, the most significant enrichment was for structural components of ribosomes. Concerning biological processes, the response to chitin and glutathione metabolism were the most common ones (Figure 5A). In *SALK-047877* and *SALK-083115*, the cellular component analysis showed that the most significant enrichment of DEGs comprised the structural constituents of ribosomes, followed by the plasma membrane, plasmodesmata, and nucleolus (Figure 5B,C). Regarding molecular functions, rRNA binding was significantly enriched (Figure 5B,C). In terms of biological processes, response to chitin and translation were significantly overrepresented items (Figure 5B,C). Under normal conditions, GO enrichment analysis of DEGs identified by comparing WT and *gun1*, *SALK_047877*, and *SALK-083115*, revealed that heme binding, quercetin 7-O-glucosyltransferase activity, and quercetin 3-O-glucosyltransferase activity were all enriched in the top ten terms (Figure S4). The KEGG enrichment analysis and visualization were performed using OECloud tools (https://cloud.oebiotech.cn, accessed on 5 March 2023). KEGG analyses were performed using RNA-seq data from: *gun1*_LIN vs. WT_LIN; *SALK_047877*_LIN vs. WT_LIN; *SALK_083115*_LIN vs. WT_LIN. According to the results of DEGs of KEGG enrichment analysis, the top 20 pathways with the lowest *p*-value (the most significant enrichment) were selected for display. In *gun1*, all the top 20 most significantly enriched pathways were divided into genetic information processing, and metabolism (Figure 5D). Of these, only the ribosome was the representative pathway in genetic information processing (Figure 5D). Notably, the remaining 19 pathways involved in metabolism included linoleic acid metabolism, photosynthesis, biotin metabolism, fatty acid, amino acid metabolism, starch, and sucrose metabolism (Figure 5D). In *SALK-047877* and *SALK-083115*, all the top 20 most significantly enriched pathways were divided into cellular processes, environmental information processing, genetic information processing, and metabolism (Figure 5E,F). Autophagy and plant hormone signal transduction were representative pathways in cellular processes and environmental information processing (Figure 5E,F). Ribosome, DNA replication, base excision repair, and homologous recombination were the main pathways in genetic information processing (Figure 5E,F). The remaining pathways included linoleic acid metabolism, isoquinoline alkaloid biosynthesis, photosynthesis-antenna proteins, fatty acid, and amino acids metabolism, and pyrimidine and purine metabolism (Figure 5E,F). Under normal conditions, KEGG enrichment analysis of DEGs, identified by the comparison between WT, and *gun1*, *SALK_047877*, *SALK-083115* shared twelve terms in the top twenty terms, including MAPK signaling pathway-plant, flavonoid biosynthesis, cutin, suberine, and wax biosynthesis, isoquinoline alkaloid biosynthesis, phenylpropanoid biosynthesis, and glutathione metabolism (Figure S3A–C). The Venn analysis indicated a substantial overlap of DEGs among the *gun1*_LIN vs. WT_LIN, *SALK_047877*_LIN vs. WT_LIN, *SALK_083115*_LIN vs. WT_LIN, with a notable count of 7238 genes (Figure S7A). This substantial overlap underscores a potentially significant commonality in the genetic response across these mutants. The GO enrichment analysis conducted on these overlapping DEGs unveiled a functional convergence in several key biological themes. Notably, the genes are significantly enriched in terms related to the “plasma membrane”, highlighting their role in cellular communication and transport (Figure S7B). Additionally, there is a substantial representation of genes associated with the “structural constituent of ribosome”, which points to their potential roles in ribosome (Figure S7B). Furthermore, the enrichment in “chloroplast”-related terms suggests a connection to photosynthesis and other chloroplast functions, indicating the significance of *AT5G08670* in the regulation of retrograde signaling (Figure S7B).

### 2.6. GO Enrichment Analysis of Plastid-Related DEGs

To evaluate the effect of LIN treatment on the transcript level of chloroplast- and mitochondria-related genes in both mutant and WT seedlings, the DEGs between LIN and control were identified. To uncover how LIN treatment affects metabolic and biosynthetic processes related to chloroplasts and mitochondria, and whether these effects differ between mutants (*gun1*, *SALK_047877*, *SALK_083115*) and WT plants, the GO enrichment analysis was carried out. GO enrichment analysis indicated that majority down-regulated mitochondria-related DEGs in *gun1*, *SALK_047877*, and *SALK_083115* were involved in the generation of precursor metabolites and energy, carbon metabolism, NADH, and amino acid metabolic processes (Figure S4A). LIN treatment appeared to suppress these vital mitochondrial functions, leading to reduced metabolic activity in the mitochondria. This suppression is uniformly observed in *gun1*, *SALK_047877*, and *SALK_083115* mutants, indicating a shared response to LIN-induced stress affecting these pathways. Up-regulated mitochondria-related DEGs in *SALK_047877* and *SALK_083115* primarily participated in protein targeting to mitochondrion, protein folding, mitochondrion organization, and mitochondrial gene expression (Figure S4B). Conversely, GO enrichment term was similar in WT and *gun1*, involved in valine, leucine, and isoleucine degradation, amino acid catabolism, mitochondrial transmembrane transport, and reactive oxygen species (Figure S4B). Down-regulated chloroplast-related DEGs were enriched in comparable GO terms in *gun1*, *SALK_047877*, *SALK_083115*, and WT seedlings, including the chlorophyll metabolic process, biosynthesis of amino acids, starch metabolic processes, photosynthesis, and carbon metabolism (Figure S4C). Notably, sulfate assimilation was inhibited exclusively in mutants, and protein import into chloroplast stroma was only inhibited in WT (Figure S4C). The down-regulation of these processes highlights a reduction in chloroplast functionality, impacting photosynthetic efficiency and metabolic activities. Up-regulated chloroplast-related DEGs were enriched in similar GO terms in plastid organization, protein localization to chloroplast, heterocycle biosynthetic process, and photosynthesis (Figure S4D). In *SALK_047877* and *SALK_083115* mutants, DEGs were enriched in ribosomal processes (Figure S4D). In *gun1* and WT, DEGs were enriched in response to oxidative stress, regulation of photosynthesis, and carbon metabolism (Figure S4D). These findings delineate a complex interplay between LIN-induced stress and the cellular responses in chloroplasts and mitochondria.

### 2.7. Expression of Nuclear Genes of Mitochondrial Proteins Is Affected by the Loss of AT5G08670

**Figure 6:**
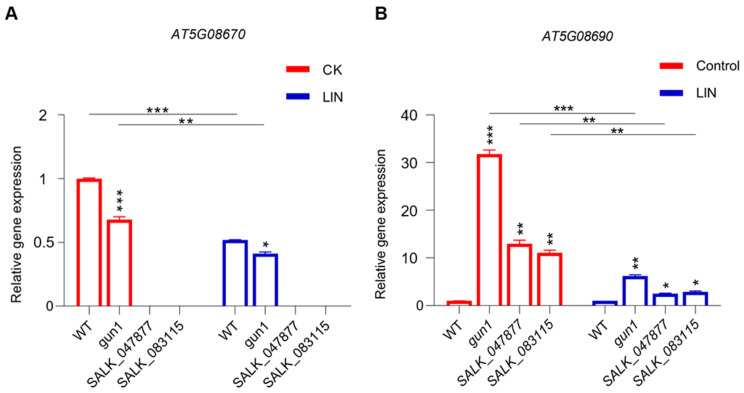
Analysis of the expression of *AT5G08670* and *AT5G08690*. (**A**) *AT5G08670* expression analysis in WT and mutants in control and with LIN treatment, respectively. (**B**) Analysis of *AT5G08690* expression in wild-type (WT) and mutants with and without LIN treatment. Gene expression levels were evaluated by RT-qPCR, *UBQ7* was used as a reference gene. Significant differences are indicated by asterisks (one–way ANOVA with Tukey’s multiple comparisons test of mutants vs. WT, and * *p* < 0.05, ** *p* < 0.01, *** *p* < 0.001, *n* = 3).

To confirm that *AT5G08670* is involved in mitochondrial signaling, RT-qPCR was performed to check the expression level of *AT5G08670* and *AT5G08690*, under normal conditions (CK) and LIN treatment. From the RT-qPCR data, we found that the expression levels of these two genes are different under normal conditions and LIN treatment. As expected, *AT5G08670* expression was undetectable under any condition in the SALK lines (Figure 6A), while the expression level of *AT5G08690* was significantly higher in the SALK lines compared to the WT under normal conditions (Figure 6B). Similarly, after LIN treatment, the expression level of *AT5G08690* was still significantly higher in the SALK lines compared to the WT, despite LIN inhibiting *AT5G08690* expression in mutants (Figure 6B). Here, *gun1* was used as an experimental control which shows a similar gene expression pattern of *AT5G08670* as WT but a strong increase of expression of *AT5G08690*. Taken together, these results indicate that changes in mitochondrial ATP synthase activity affect retrograde signaling in the absence of *AT5G08670*.

**Figure 7:**
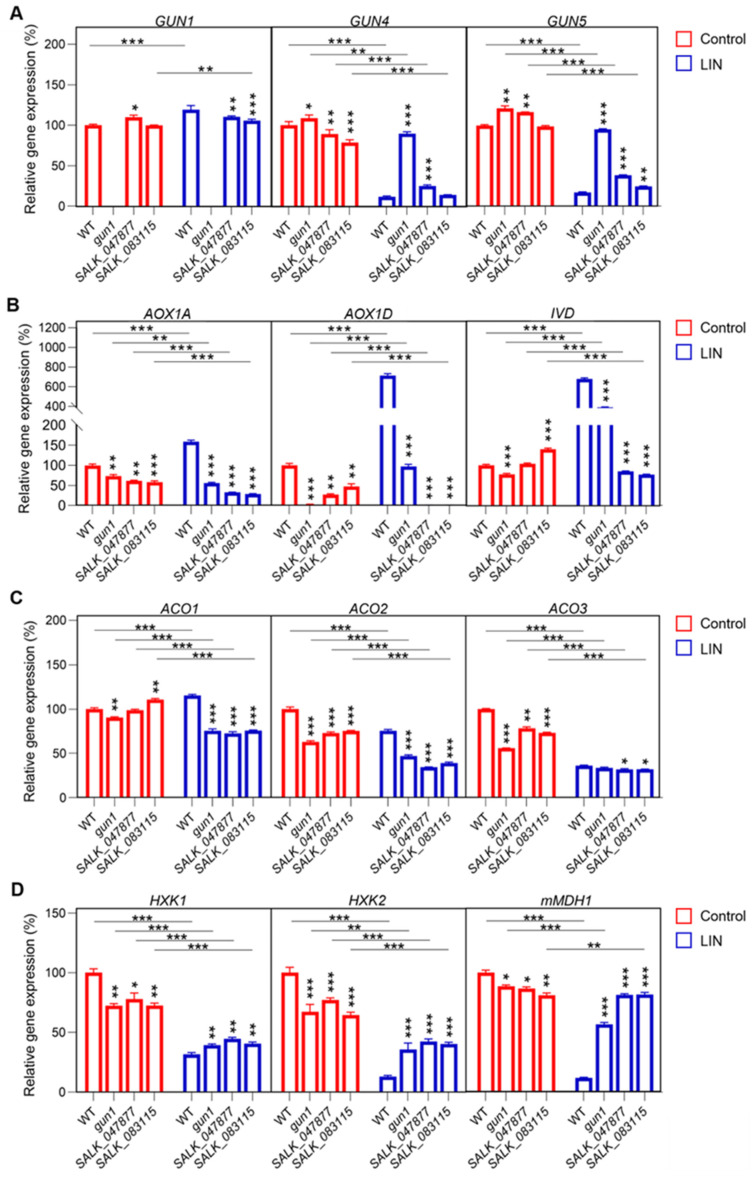
Expression analysis of nuclear genes of mitochondrial and chloroplast proteins. (**A**) Analysis of the expression of chloroplast-related genes. (**B**) Analysis of the expression of mitochondrial-related genes. (**C**,**D**) Analysis of the expression of carbon metabolism-related genes. Significant differences are indicated by asterisks (one–way ANOVA with Tukey’s multiple comparisons test, * *p* < 0.05, ** *p* < 0.01, and *** *p* < 0.001, *n* = 3).

We next analyzed the effects of *AT5G08670* on some key genes involved in plastid and mitochondrial signaling. *GUN1*, *GUN4*, and *GUN5* are important for plastid signaling. After LIN treatment, the expression of *GUN4* and *GUN5* was decreased to different degrees in the T-DNA insertion mutants, in which the expression of *GUN1* increased relative to the control (Figure 7A). We also analyzed the expression of important nuclear genes of mitochondrial proteins such as *AOX1A*, *AOX1D*, and *ISOVALERYL-COA-DEHYDROGENASE* (*IVD*). The expression of *AOX1A*, *AOX1D*, and *IVD* increased in WT seedlings treated with LIN, and the expression of these genes was decreased in *SALK_047877* and *SALK_083115* (Figure 7B). Further, the analysis of the expression of carbon metabolism genes revealed that *1-Aminocyclopropane-1-Carboxylate Oxidase 1* (*ACO1*) was decreased in all groups, except in WT seedlings following LIN treatment; the expression of *ACO2*, *ACO3*, *Hexokinase 1* (*HXK1*), and *HXK2* was decreased to different degrees in all samples (Figure 7C,D), and the expression of *mitochondrial Malate Dehydrogenase 1* (*mMDH1*) was significantly inhibited in WT seedlings (Figure 7D). The transcript levels of LIN-repressed genes, such as *GUN4*, *GUN5*, *HXK1*, *HXK2*, and *mMDH1*, were higher in *gun1*, *SALK_047877*, and *SALK_083115* seedlings than in WT seedlings treated with LIN. In contrast, the transcript levels of *AOX1D*, *AOX1A*, *ACO1*, and *ACO2* were lower in *gun1*, *SALK_047877*, and *SALK_083115* seedlings than in WT seedlings in the presence of LIN. These findings indicate that *GUN1* and *AT5G08670* dependent signaling pathways play important roles in the expression of nuclear genes of both chloroplast and mitochondrial proteins in response to LIN.

### 2.8. Identification of Important TFs Downstream of AT5G08670-Involved Signaling

**Figure 8:**
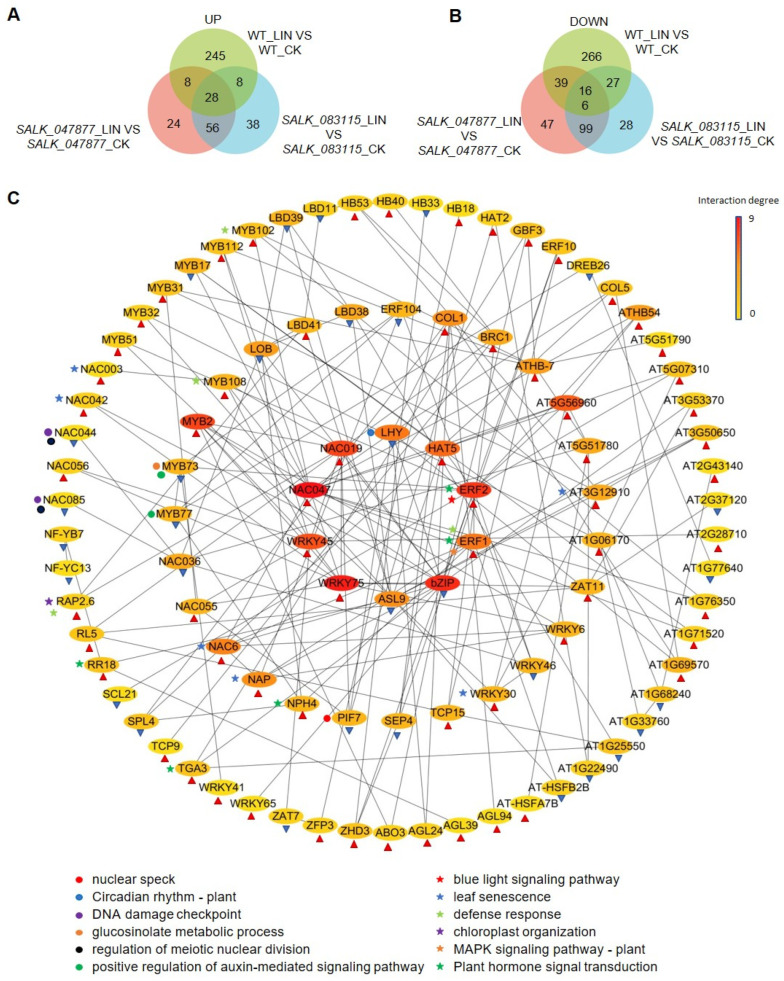
Analysis of the TF network of DEGs between the *AT5G08670* SALK lines and WT. (**A**,**B**) Venn diagram analysis of significantly down/up-regulated TFs in the presence of LIN. (**C**) Diagram of the interaction network of TFs. The network was created by STRING and Cytoscape. The color key indicates low to high interaction strength. The genes only up-regulated in *AT5G08670 SALK* lines are denoted by blue triangles. The genes which are down-regulated in *AT5G08670 SALK* lines are denoted by red triangles. Circles indicate GO and KEGG item entries enriched in up-regulated differentially expressed TFs; pentacles indicate GO and KEGG item entries enriched in down-regulated differentially expressed TFs.

Differentially expressed genes of transcription factors in the presence of LIN were ordered into up-regulated and down-regulated groups. Venn diagrams (Figure 8A) were plotted for the three groups of up-regulated transcription factors. The results showed that there were 28 combing shares of up-regulated genes in *SALK_047877*, *SALK_083115*, and WT, and 56 up-regulated genes in the in *SALK_047877* and *SALK_083115* (Figure 8A, Table S6). Venn diagrams (Figure 8B) were performed for the three groups of down-regulated genes. The results showed that there were 99 down-regulated genes in *SALK_047877* and *SALK_083115* (Figure 8B, Table S7). Based on these differently expressed TFs, we constructed an interaction network of TFs to characterize the downstream network associated with *AT5G08670*-dependent signaling. Analysis of the protein-protein interaction network revealed that LHY, PIF, MYB, WRKY, and ERF may play key regulatory roles in *AT5G08670*-dependent signaling (Figure 8C). In *Arabidopsis* photosynthesis and carbohydrate metabolism, the LHY is epigenetically repressed, leading to the upregulation of *GI* (*GIGANTEA*), and *EE (EVENING ELEMENT)* downstream genes, which altered circadian rhythms and plant growth development [35]. During photosynthesis, the phytochromes are encoded by a small gene family of five members, *PHYA* to *PHYE*; the *PHYA* is light-labile, whereas *phyB* to *phyE* are more light-stable [36]. *PIF* (*Phytochrome-Interacting Factor*) was the first member of the bHLH family identified as a specific interactor of light-activated *PHYA* and *PHYB* [37]. The MYB TF family contains 200 genes and is the largest TF family in *Arabidopsis*, accounting for 9% of all the TFs in this plant [38,39]. Many members of the MYB TF family play a role in tolerance to abiotic stress [7]. The subset of these genes includes many transcription factors (TFs) like WRKY, ERF, NAC, and MADS. WRKYs are of particular interest as they are involved in diverse biotic/abiotic stress responses as well as in developmental/physiological processes [40]. WRKYs are also regulated through inter-organelle retrograde signaling. An example of mitochondrial retrograde regulation involves plant *NDPKs* (nucleoside diphosphate kinases) that are involved in stress response, hormone response, and light signaling [41]. *AtWRKY53* has diverse roles and its expression is tightly regulated [42]. Furthermore, AP2/ERF family transcription factors have emerged as key regulators of several abiotic stresses and respond to multiple hormones [43]. Members of the AP2/ERF superfamily transcription factors (TFs) play a crucial role in chloroplast division during NaCl stress [44]. ERF TFs are the largest family in this superfamily and participate in many developmental and stress response processes in plant cells.

Through GO and KEGG enrichment analysis, the up-regulated genes in WT were enriched in those involved in the mitogen-activated protein kinase (MAPK) signaling pathway, auxin-activated signaling pathway, ethylene-activated signaling pathway, and plant hormone signal transduction. The unique down-regulated genes in WT were enriched in those involved in circadian rhythm, response to blue light, response to abscisic acid, nucleotide-excision repair, and plasmodesmata-mediated intercellular transport (Figure 8C). The MAPK signaling pathway, auxin-activated signaling pathway, and the ethylene signaling pathway are important to plants for responding to adverse environmental conditions [45]. In the SALK lines, the *AT5G08670*-dependent retrograde signal is absent, making the associated signal pathways unresponsive. Plastid retrograde signals regulate many biological processes, such as the circadian clock. The interaction between the circadian clock and chloroplast retrograde signaling systems could regulate transcription-translation feedback loops to adapt to environmental changes [46]. These results show that certain TFs in mitochondria, chloroplasts, and nuclei might be involved in *AT5G08670*-dependent signaling to control plastid retrograde signaling.

## 3. Discussion

### 3.1. AT5G08670: An Impact on PhANG Expression and Its Operational Consequences

Utilizing mitochondria and chloroplasts is essential for plant growth and development. Plastid development is regulated by the nucleus but also affects nuclear transcriptional activity through plastid feedback by retrograde signaling [47,48]. Mitochondrial development is also affected by chloroplasts. In rice, a single chloroplast mutation impairs mitochondrial development [49]. Therefore, mitochondrial gene defects might also affect chloroplast development. *AT5G08670* encodes an ATP synthase *beta*-subunit in mitochondria. The mitochondrial *beta-*subunit is encoded by three genes: *AT5G08670*, *AT5G08680*, and *AT5G08690* (Figure 1A). *AT5G08670* and *AT5G08690* are expressed, whereas *AT5G08680* is not according to the GUS assay results, and they encode an identical mature protein (Figure 1C), implying functional redundancy between these two genes.

The plastid retrograde signaling pathway has been extensively studied, and several mutants impaired in this pathway have been identified in *Arabidopsis*, such as *gun1*, *gun4*, *coe1*, and *coe2* [6,7,9,11]. PhANGs are significantly down-regulated in chloroplasts under biotic or abiotic stress, and the normal connection between the chloroplast and nuclear genome is compromised in mutants affected by retrograde signaling [8]. From our RT-qPCR analysis, we found that when seedlings were treated with LIN and NF, the expression of Light-Harvesting Chl a/b-Binding (LHCB) genes (*LHCB1.2* and *LHCB2.1*) was significantly higher in *gun1*, *SALK_047877* and *SALK_083115* than in WT (Figure 2C). In the control plants without treatment, *LHCB1.2* expression in the *AT5G08670* mutant remains at levels similar to those in WT plants. In contrast, the expression of *LHCB2.1* is notably reduced in the *AT5G08670* mutant compared to WT (Figure S6). These results suggest that *AT5G08670* may play a role in plastid retrograde signaling. Transcriptome analysis of the *AT5G08670* mutants revealed that *AT5G08670* is implicated in retrograde signaling, which in turn affects the expression of nuclear genes encoding both mitochondrial and chloroplast proteins (Figure 7) raising the possibility that *AT5G08670* might be involved in both plastid and mitochondrial retrograde signaling transduction although the mechanisms of mitochondrial regulation of chloroplast development are unknown. However, in maize mutants with defective mitochondrial genes [50,51], the leaves show yellow or pale green stripes indicating that mitochondria affect the development of chloroplasts.

### 3.2. Mitochondrial Proteins Contribute to Plastid Retrograde Signaling

The *gun* mutants are the best-characterized materials for plastid retrograde signaling. Whereas *GUN1* encodes a chloroplast nucleoid pentatricopeptide repeat protein [9], the other GUN proteins are involved in the tetrapyrrole biosynthetic pathway. *GUN2* encodes a heme oxygenase [10], *GUN3* encodes a photosensitive pigment chromophore synthase, *GUN4* encodes a protein involved in chlorophyll synthesis [8,11], and *GUN5* encodes the H subunit of magnesium chelatase [12]. *GUN1* might also play a role in the tetrapyrrole pathway [52]. Because these GUN proteins are located in the chloroplast, they play a direct role in the retrograde chloroplast signaling pathway [53]. However, little is known regarding the roles of mitochondrial proteins in affecting plastid retrograde signaling.

**Figure 9:**
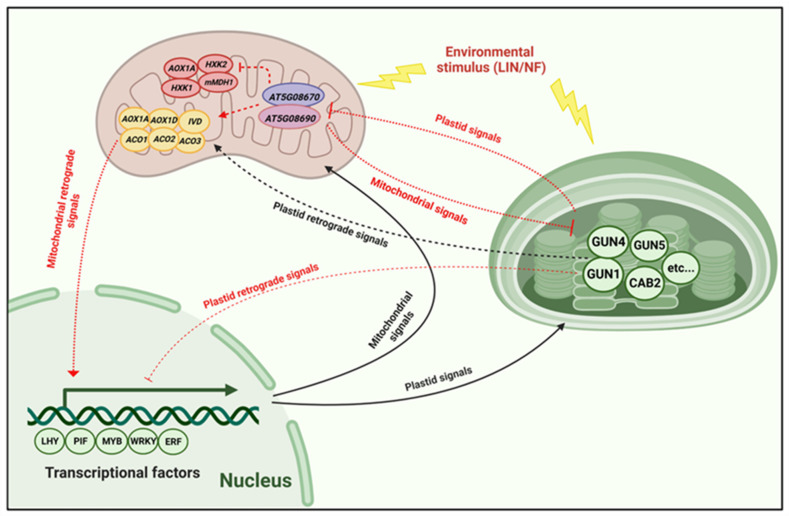
A schematic presentation of retrograde signaling in plant cells based on our study. Environmental factors in this study are the chloroplast development inhibitors LIN and NF, which affect chloroplast and mitochondria (shown by the yellow lightning symbol) and generate photo-oxidative damage under high light. The signal generated by the chloroplast is transduced to the nucleus (shown by a red line), causing the necessary changes in NGE (plastid retrograde signaling). The “T” arrow represents “inhibition”; and the arrow represents “promotion”. The transcriptome analysis shows that the expression of chloroplast and mitochondrial retrograde signaling-related genes is affected in *AT5G08670* seedlings treated with LIN and associated with the expression of different TFs, such as LHY, PIF, MYB, WRKY, and ERF.

The nucleus controls the majority of processes in chloroplasts, including organelle gene expression (OGE) via ‘anterograde signaling’. The nucleus, in turn, depends on the signals originating from the chloroplasts that convey information to the nucleus via ‘retrograde signaling’. This system allows for changes in nuclear gene expression (NGE) in response to the status of the chloroplast. We propose a model from our study in which environmental cues affect the chloroplast and mitochondrial state which in turn gives rise to retrograde signals that alter nuclear gene expression from the transcriptional to the post-translational level and ultimately feedback to plastid function (Figure 9). Initially, the environmental stimulus is perceived by the chloroplast. In this study, environmental factors include LIN, NF, and high light (shown by the yellow arrows) (Figure 9). The compensation effect between *AT5G08670* and *AT5G08690* is an intriguing phenomenon. When the function of *AT5G08670* is lost, a significant increase in the expression of the cognate gene *AT5G08690* is observed (Figure 6B). This suggests a compensatory mechanism at play, potentially to maintain certain cellular processes or functions that are otherwise disrupted by the loss of *AT5G08670*. This compensation effect bears resemblance to that seen between *bZIP25* (*basic-region leucine zipper 25*) and *bZIP53* (*basic-region leucine zipper 53*) [54,55]. Just as in that case, it implies a regulatory network within the cell that strives to restore balance and functionality when one component is compromised. It may involve factors such as shared regulatory elements, feedback loops, or cross-talk between different signaling pathways. After LIN treatment, the expression of *GUN4* and *GUN5* was decreased to different degrees in the SALK mutants and WT (Figure 7A). LIN triggers retrograde signals in chloroplasts to regulate the expression of relevant nuclear genes coding for specific chloroplast proteins [56]. Compared to the SALK mutants and WT, *gun1* is insensitive to LIN. We also analyzed the expression of nuclear genes of important mitochondrial proteins like *AOX1D*, *AOX1A*, and *IVD*. The expression of *AOX1D* and *AOX1A* increased in WT seedlings treated with LIN, and the expression of these genes was decreased in *SALK_047877* and *SALK_083115* (Figure 7B). Plants have developed a variety of strategies to reduce the formation of hazardous ROS that arise during environmental changes [57]. One of them is AOX-mediated alternative respiration which decreases production of toxic ROS [58]. *AOX1A* is a marker gene of mitochondrial retrograde regulation [59]. Stress induces mitochondrial retrograde signaling, which promotes *AOX1A* expression by activating the *NAC017* signaling pathway [50]. With salt stress, *MYB30* promotes *AOX1A* expression and AOX-mediated alternative respiration to decrease toxic ROS accumulation, as well as *AOX1D* [51]. We, therefore, propose that *AT5G08670* affects *AOX1A* and *AOX1D* expression to improve the tolerance to LIN via mitochondrial retrograde signaling. Cells require more energy to maintain their normal physiological activities with LIN stress [60]. *IVD* is a key enzyme involved in the process of leucine catabolism and catalyzes the dehydrogenation of isovaleryl-CoA to *beta*-methylcrotonyl-CoA while transferring the electrons to electron-transferring flavoprotein (ETF) for the synthesis of ATP to survive [9,61,62]. Further, analysis of the expression of genes involved in carbon metabolism revealed that *1-Aminocyclopropane-1-Carboxylate Oxidase 1* (*ACO1*) was decreased in all groups, except in WT seedlings following LIN treatment; the expression of *ACO2*, *ACO3*, *hexokinase 1* (*HXK1*), and *HXK2* was decreased to different degrees in all samples (Figure 7C,D). It is possible that LIN caused damage to mitochondria and chloroplasts, which inhibited photosynthesis and respiration. As a result, the expression of genes related to carbon metabolism was decreased to maintain a lower consumption level. Maybe this response represents a survival strategy in the face of adversity. For example, hibernation allows for a low metabolism that can sustain life when no food is available [63]. Furthermore, we examined some marker genes (*AT5G46710*, *MZA15.12*, *AT4G37910*, *HSP70-9*, *AT1G20950*, *F9H16.6*, *AT4G36250*, and *ALDH3F1*) involved in mitochondrial dysfunction, chloroplast ROS, and PAP signaling pathways (Figure S5), which follow the same gene expression pattern as carbon metabolism genes (Figure 7D). *MZA15.12* is a member of the PLATZ (plant AT-rich protein and zinc-binding protein) transcription factor family, a class of plant-specific zinc-dependent DNA-binding proteins. PLATZ is important for seed endosperm development and cell proliferation during the early stages of crop development. The *HSP70-9*, *F9H16.6*, and *ALDH3F1* genes encode ATP-binding unfolding proteins that respond to zinc, different stress hormones, and sucrose in the mitochondrion. All three of these genes regulate 3-chlorophyll aldehyde dehydrogenase activity [64,65].

In summary, these findings indicate that altered expression of *GUN1* and *AT5G08670* affects retrograde signaling which plays an important role in the expression of nuclear genes of both chloroplast and mitochondrial proteins in response to LIN (Figure 9). LIN treatment induces significant transcriptional reprogramming across both organelles, revealing distinct yet overlapping stress adaptations among different genotypes. This study deepens our understanding of how LIN disrupts plant cellular functions and offers insights into broader mechanisms of stress tolerance and adaptation.

Identifying TFs downstream of the *AT5G08670*-involved signaling pathway offers new insights into the complex regulatory networks within plant cells. Our study offers an extensive overview of the interaction network of differentially expressed TFs in response to LIN treatment (Figure 8, Tables S6 and S7). The unique down-regulation of 99 TFs in the mutants indicates a significant shift in the transcriptional landscape (Figure 8B), potentially leading to phenotypic changes observed in mutant lines. This down-regulation might represent a compensatory mechanism to adjust for the loss of *AT5G08670* function, suggesting a broader role of this gene in maintaining cellular homeostasis. MYB-related transcription factors play a crucial role in chloroplast biogenesis in *Marchantia polymorpha* and *Arabidopsis thaliana* [66]. Mutations in MYB-related genes in these plants lead to severely impaired chloroplast development and substantial disturbances in the expression of genes essential for photosynthesis [66]. Recent studies have highlighted the regulatory roles of PIFs in chloroplast development [67]. *MYB30* promotes the accumulation of *PIF4* and *PIF5* proteins under light conditions [68]. WRKY transcription factors act as crucial mediators between chloroplast and mitochondrial disruptions [69]. The four WRKY TFs identified as affecting transcripts encoding mitochondrial proteins were examined for a role in the expression of genes encoding chloroplast proteins, highlighting a coordinated regulatory network for stress-responsive genes in both organelles [70]. The *Arabidopsis* NAC domain transcription factor *NAC017* is recognized as a regulator of the mitochondrial retrograde response, linking mitochondrial stress with nuclear gene regulation [71]. Studies have also emphasized the importance of retrograde signaling from mitochondria, which activates *NAC017*, a transcriptional activator of *AOX1a* during mitochondrial dysfunction [72]. *NAC013* mediates mitochondrial retrograde regulation in response to oxidative stress by interacting with the mitochondrial dysfunction motif (MDM), thereby enhancing oxidative stress tolerance in *Arabidopsis* [73]. Our findings underscore the multifaceted role of *AT5G08670* in regulating the expression of TFs that are critical for plant adaptation and development. The identification of these TFs lays the groundwork for further studies aimed at elucidating the molecular mechanisms underlying the crosstalk between mitochondria and plastid retrograde signaling.

The level of ATP synthase *beta*-subunit and the total ATP synthase activity in the mutants were significantly lower than in WT (Figure 3A,B). It is thus likely that the effects observed are due to changes in cellular ATP, indicating that perturbation of ATP homeostasis in mitochondria affects not only mitochondrial but also chloroplast metabolism and retrograde signaling. Interactions between chloroplast and mitochondrial ATP metabolism have also been observed in Chlamydomonas in a suppressor strain of a chloroplast mutant lacking the *atpB* gene. This suppressor strain was able to grow photoautotrophically in the absence of chloroplast ATP synthase [74,75]. In this strain, photosynthesis was sensitive to specific inhibitors of mitochondrial electron transport suggesting that photosynthesis was restored through an unusual interaction between mitochondria and chloroplasts involving the export of reduced compounds from the chloroplast to mitochondria to stimulate the synthesis of mitochondrial ATP which in turn would be exported from the mitochondria to the chloroplast. Interestingly, a role in extracellular ATP signaling and regulation of cell death has been shown for the *AT5G08690* gene [54] and adds further support for an important function of the b subunit of ATP synthase in signaling pathways. It remains to be determined how ATP levels are sensed in mitochondria and chloroplasts and how perturbations in ATP homeostasis in either organelle are compensated.

## 4. Materials and Methods

### 4.1. Plant Materials and Growth Conditions

All *Arabidopsis* strains used in this study were in the Columbia (Col-0) ecotype background and were obtained from the Arabidopsis Biological Resource Center. Homozygous mutants were identified by PCR with corresponding primers. All mutants and WT seedlings were grown in an artificial climate chamber with the following growth conditions: 21–23 °C, 100 μmol photons m^−2^ s^−1^, 18-h light/8-h dark cycles, and 60–70% humidity. For the NF and LIN treatments, surface-sterilized mutants and WT seeds were planted on 1/2 Murashige and Skoog (1/2 MS) medium (PhytoTechnology Laboratories, LLC™, Lenexa, KS, USA) containing 1% sucrose and 0.8% agar supplemented with either 5 µM NF (Sandoz Pharmaceuticals; Vienna, Austria) or 220 µg/mL LIN (Sigma; St. Louis, MO, USA).

### 4.2. DNA Extraction

Fresh seedlings (0.1 g) were cut, wrapped in tinfoil, and quick-frozen in liquid nitrogen barrels. The quick-frozen samples were then placed into a mortar and ground quickly and thoroughly with a pestle. Next, 650 µL of preheated cetyltrimethylammonium bromide (CTAB) buffer was added and mixed with the ground samples; the same amount of chloroform was then added, and the contents were mixed slowly. After centrifugation at 12,000× *g* for 15 min, the supernatant of the liquid was transferred to a 1.5 mL Eppendorf (EP) tube, the same amount of pre-cooled isopropyl alcohol on ice was added and mixed by slowly inverting the EP tube. The supernatant was removed after centrifugation at 12,000× *g* and 4 °C for 10 min, 1 mL of 70% ethanol was added to the EP tube to remove the liquid supernatant, and the precipitated DNA was recovered by immersing the EP tube in ethanol solution. Centrifugation was performed at 12,000× *g* and 4 °C for 10 min, the supernatant was discarded, and this procedure was repeated (i.e., a total of two rounds of centrifugation). The EP tube was left open for several minutes to dry the adsorption column at room temperature; 50 µL of sterile water was then added to dissolve the precipitated DNA and stored at −20 °C.

### 4.3. RNA Extraction and Quantitative Real-Time PCR (RT-qPCR)

Total RNA was extracted from 80–100 mg of frozen, homogenized *Arabidopsis* tissue using the MagMAX Plant RNA Isolation Kit (Applied Biosystems, Foster City, CA, USA) following the manufacturer’s instructions. cDNA was synthesized using the NovoScript Plus All-in-one 1st Stand cDNA Synthesis, SuperMix Kit (Novoprotein, Shanghai, China). RT-qPCR was performed using the NovoStart SYBR qPCR SuperMix Kit (Novoprotein, Shanghai, China) in a QuantStudio TM 12K Flex Real-Time PCR system (Applied Biosystems, Foster City, CA, USA). The thermal cycling conditions were as follows: 95 °C for 2 min; 40 cycles at 95 °C for 20 S; and 60 °C for 30 S. The primers are shown in Table S8. Data were analyzed using QuantStudio TM 12 K Flex software version 1.2.3 (Applied Biosystems, Foster City, CA, USA). Significant differences were evaluated using Student’s *t*-test, and asterisks indicate significant *p*-values.

### 4.4. RNA Sequencing Analyses

WT, *SALK_047877*, *SALK_083115*, and *gun1* were cultured on 1/2 MS (with/without 220 µg/mL LIN) for one week, with three replicates. Total RNA was extracted using the mirVana miRNA Isolation Kit (Ambion, Austin, TX, USA) following the manufacturer’s protocol. The integrity of RNA was assessed using an Agilent 2100 Bioanalyzer (Agilent Technologies, Santa Clara, CA, USA). Samples with an RNA integrity index greater than 7 were used in subsequent analyses. Libraries were constructed using the TruSeq Stranded mRNA LT Sample Prep Kit (Illumina, San Diego, CA, USA) following the manufacturer’s protocol. Sequencing of these libraries was then conducted on an Illumina sequencing platform (HiSeqTM 2500 or Illumina HiSeq X Ten), and 125 bp/150 bp paired-end reads were generated. The number of counts for each gene was normalized using DESeq2 software version 3.14. The negative binomial distribution test was performed to determine the multiplicity of differences in data, estimate expression, and evaluate the significance of reading differences using the base mean values. A gene was considered to be differentially expressed by applying a threshold of 0.05 for the *p*-value and 1 for the log_2_FC. Additionally, we filtered away genes that had very low counts, and specifically, we did not consider a gene expressed if it did not exceed in at least one condition an average read count of 10 in the samples. We used Trimmomatic software version 0.39 [76] for quality preprocessing of the original data in light of the impact of data error rate on the outcomes, and statistically summarized the total number of reads during the quality control procedure. To locate reference genomes or genes and learn specifics about the sequencing features of sequenced data, we used hisat2 [77] to align Clean Reads with chosen reference genomes. By counting the number of reads that were found in the exonic parts of the protein-coding genes, the levels of expression of those genes were determined. Using a database of known reference gene sequences and annotation files, a method called sequence similarity matching was used to determine quantitatively the expression of each protein-coding gene in each sample. The number of reads that were aligned to protein-coding genes in each sample was determined using HTSeq-count software version 0.13.5 [78], and the fragments per kilobase of transcript (FPKM) value of protein-coding gene expression was determined using this parameter to measure gene expression levels [79].

### 4.5. Protoplast Transient Expression Assay

We isolated protoplasts from the cotyledons of one-week-old Arabidopsis seedlings using the method previously described [38]. The middle part of a leaf was cut into 0.5–1-mm leaf strips which were submerged in the enzyme solution. Leaf strips were vacuum-infiltrated for 30 min and incubated at room temperature on a shaker for 3 h in the dark. Released protoplasts were filtered and resuspended at a concentration of 1 × 10^6^ cells/mL. To construct the AT5G08670-GFP fusion expression plasmids, the entire coding sequence (CDS) was inserted into the pCambia2300 vector with the ClonExpress II One Step Cloning Kit (C112-01, Vazyme, Nanjing, China). The recombinant plasmid was then transformed into *E. coli*, DH5 alpha strain for amplification. 105 protoplasts and 20 µg plasmids were mixed and incubated at 25 °C for 20 h. Then, GFP fluorescence was observed in a confocal laser microscope.

### 4.6. GUS Staining and Histological Analysis

To analyze the organ specificity of the expression patterns of *AT5G08670* and *AT5G08690*, we generated a pro*AT5G08670*:GUS and pro*AT5G08690*:GUS construct consisting of a 2 kb fragment of the *AT5G08670* and *AT5G08690* promoter to drive the GUS reporter gene. The pro*AT5G08670*:GUS and pro*AT5G08690*:GUS constructs were transformed into the WT (Col-0) background. Histochemical GUS staining was performed with a GUS Staining Kit (G3061, Solarbio Co., Beijing, China) following the manufacturer’s instructions. Samples were fixed in 90% acetone at −20 °C, rinsed four times with 0.1 M sodium phosphate buffer (pH 7.4), and then incubated in X-Gluc solution (0.1 M sodium phosphate (pH 7.4), 3 mM potassium ferricyanide, 0.5 mM potassium ferrocyanide, and 0.5 g L^−1^ 5-bromo-4-chloro-3-indolyl-*beta*-glucuronide cyclohexilammonium salt) at 37 °C. After staining, chlorophyll was removed from the samples by incubating them in methanol; they were then mounted in a clearing solution (a mixture of chloral hydrate, water, and glycerol in a ratio of 8:2:1). Observations were made using a stereomicroscope (MZ16F, Leica Microsystems, Wetzlar, Germany) or a microscope equipped with Nomarski optics (BX51, Olympus Co., Tokyo, Japan). To characterize vascular patterns, cotyledons were fixed in a mixture of ethanol and acetic acid in a ratio of 9:1, dehydrated through a graded series of ethanol, and then mounted with a clearing solution [80].

### 4.7. Total Protein Extraction and Immunoblot Analysis

The leaves of seedlings were harvested, and total protein was prepared following the method of Sun et al. [81]. Protein concentration was determined by bicinchoninic acid-based colorimetric detection (BCA kit, Solarbio). After the measurement of protein concentration, a WT protein dilution series was used to estimate the expression level of ATPB in the mutants. For immunoblot analyses, the proteins were fractionated by sodium dodecyl sulfate-polyacrylamide gel electrophoresis (SDS-PAGE) (15% acrylamide) [82]. Proteins were then transferred to polyvinylidene difluoride membranes with a Trans-Blot SD Semi-Dry Transfer Cell (Tanon, Shanghai, China). Membranes were blocked with 5% skim milk at room temperature for 1 h. Incubated with primary antibodies in 2% skim milk in tris-bufferd saline (TBS) containing 0.1% Tween-20 (TBS-T) overnight at 4 °C then incubated for 1 h with secondary antibody. A polyclonal rabbit anti-ATPB antibody (PHY0007S, PHYTOAB, California, CA, USA) was diluted 1:1000 for use as the primary antibody. Goat Anti-Rabbit IgG H&L (HRP) (PHY6000, PHYTOAB) was diluted 1:10,000 for use as the secondary antibody. Finally, Enhanced chemiluminescence (ECL, Thermo, Norristown, PA, USA) was used for visualizing the protein bands.

### 4.8. Mitochondrial ATP Synthase Activity Assay

Mitochondrial ATP synthase activity was measured by a colorimetric assay using the ATPase activity assay kit (D799641-0050, Shenggong, Shanghai, China) following the manufacturer’s instructions. The seedlings were cultured on 1/2 MS medium for 2 weeks and roots were collected for determination of ATP synthase activity. To approximately 0.1 g of tissue, 1 mL of reagent I was added, and the mixture was homogenized in an ice bath, centrifuged at 8000× *g* for 10 min at 4 °C and the supernatant was used as the ATP synthase extract. Sample processing was according to the manufacturer’s protocol. Finally, ATP synthase activity was determined by measuring the OD at 660 nm. Each experiment was performed with three biological samples, each with three technical replicates.

### 4.9. Phylogenetic Analysis of AT5G08670

The amino acid sequences of the AT5G08670 proteins were downloaded from the TAIR website (http://www.arabidopsis.org/, accessed on 17 July 2020). We used protein BLAST (blastp) to search for homologous proteins. Protein sequences were aligned using Clustal X. The phylogenetic tree was constructed with MEGA 7 using maximum-likelihood phylogenetic analysis with 1000 bootstrap replicates.

### 4.10. Drawing of TF Network Diagram

Screening was done for differentially expressed TFs that are specifically up- and down-regulated in *SALK_047877* and *SALK_083115* after LIN treatment. Protein-protein interaction (PPI) network analysis on the string database (https://string-db.org/, accessed on 17 July 2020) was performed. The results of this analysis were exported from the string database in tab separated values (TSV file format), and annotated.

### 4.11. Generation of Complementary Lines

First, we cloned the promoter sequence and the CDS sequence of the *AT5G08670* gene. The CDS sequence of *AT5G08670* was fused to the pCAMBIA1300 vector containing MYC, without the stop codon, and driven by the promoter of *AT5G08670* for its expression. We constructed the complementary lines of *SALK_047877* using the *Agrobacterium* floral dip method. Preliminary screening was performed using MS medium containing hygromycin resistance, followed by immunoblot analysis with MYC on the obtained transgenic plants. The expected specific band is detected in the transgenic plant material, which was only identified as the complementary line.

## 5. Conclusions

This study provides evidence that the mitochondrial ATP synthase *beta*-subunit, encoded by the *AT5G08670* gene, plays a pivotal role in plastid retrograde signaling in *Arabidopsis*. Our results demonstrated that mutants affected in the *AT5G08670* gene exhibit impaired retrograde signaling, affecting the expression of *PhANGs*. Transcriptome analysis and subsequent gene expression studies highlight the intricate relationship between mitochondrial function and the regulation of nuclear genes encoding chloroplast and mitochondrial proteins. These findings suggest a model where environmental cues influence both chloroplast and mitochondrial status, leading to retrograde signals that adjust nuclear gene expression and ultimately feedback to plastid function.

Furthermore, the study revealed the potential for mitochondrial proteins to contribute to plastid retrograde signaling. The characterization of the *AT5G08670* mutants and their response to lincomycin (LIN) treatment offers insights into how mitochondrial proteins might impact chloroplast function and the broader implications for plant growth and adaptation to stress.

## Data Availability

All data supporting the findings of this study are available within the paper and within its Supplementary Data published online.

## Supplementary Materials

The following supporting information can be downloaded at: https://www.mdpi.com/article/10.3390/ijms25147829/s1.
