# Supplementary material for: Mitochondrial ATP Synthase beta-Subunit Affects Plastid Retrograde Signaling in Arabidopsis
Source: Int J Mol Sci. 2024 Jul 17;25(14):7829. doi: 10.3390/ijms25147829 (PMC11277312; doi:10.3390/ijms25147829)
Supplement: Supplementary file 1 [file ijms-25-07829-s001.zip › Supplementary Figures.pdf]

Supporting Information

Predicted proteins

GLR1_DROME
Prediction: Mitochondrial transfer peptide

CS pos: 51-52. AEY-AT. Pr: 0.7966

Protein type	Other	Signal peptide	Mitochondrial transfer peptide	Chloroplast transfer peptide	Thylakoid luminal transfer peptide
Likelihood	0.0028	0	0.9895	0.0077	0

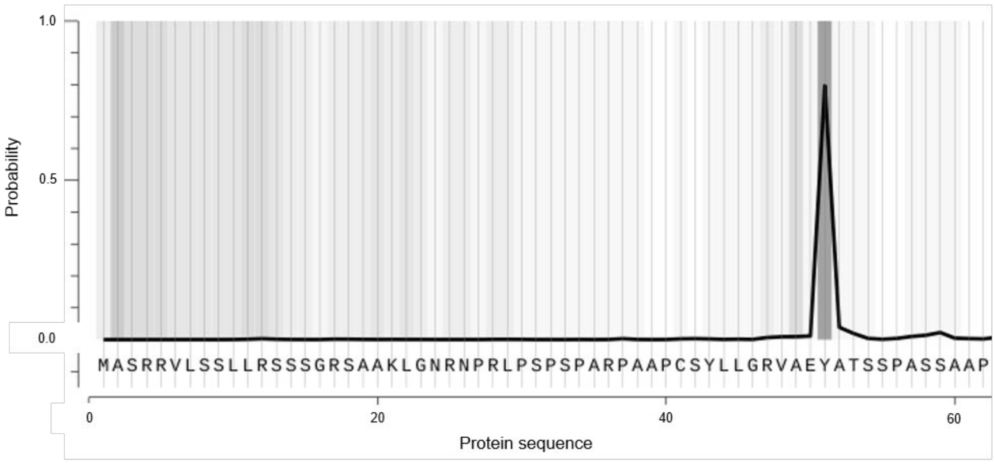

Figure S1. Leader peptide prediction of AT5G08670. Leader peptide prediction of AT5G08670 by TARGET P 2.0 shows that the cleavage site is between the 51st and 52nd amino acid.

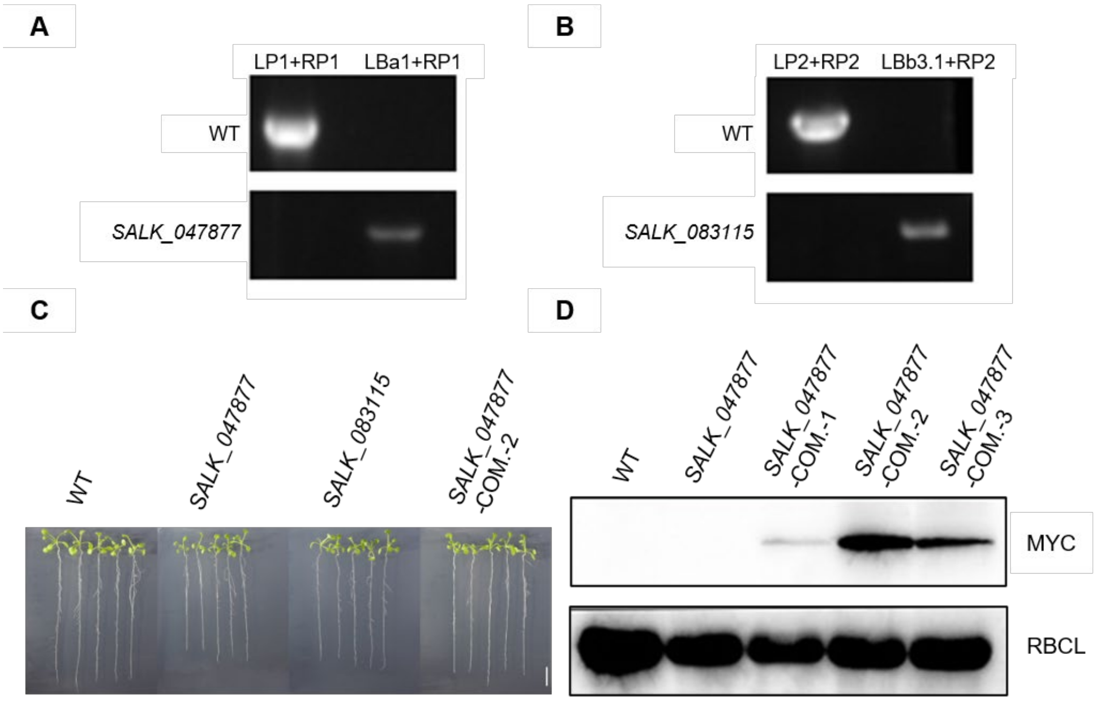

Figure S2. Identification of homozygous T-DNA insertion mutants and *SALK_047877* complementary lines. (A) Identification of homozygous *SALK_047877* with LBb3.1 T-DNA insertion primer. (B) Identification of homozygous *SALK_083115* with LBb3.1 T-DNA insertion primer. (C) Phenotype of *SALK_047877*, *SALK_083115* and a *SALK_047877* complementary line, at two weeks. Scale bar, 1 cm. (D) Detection of full-length *AT5G08670* CDS fused with a MYC-tag expression in complementary strains by Western blot.

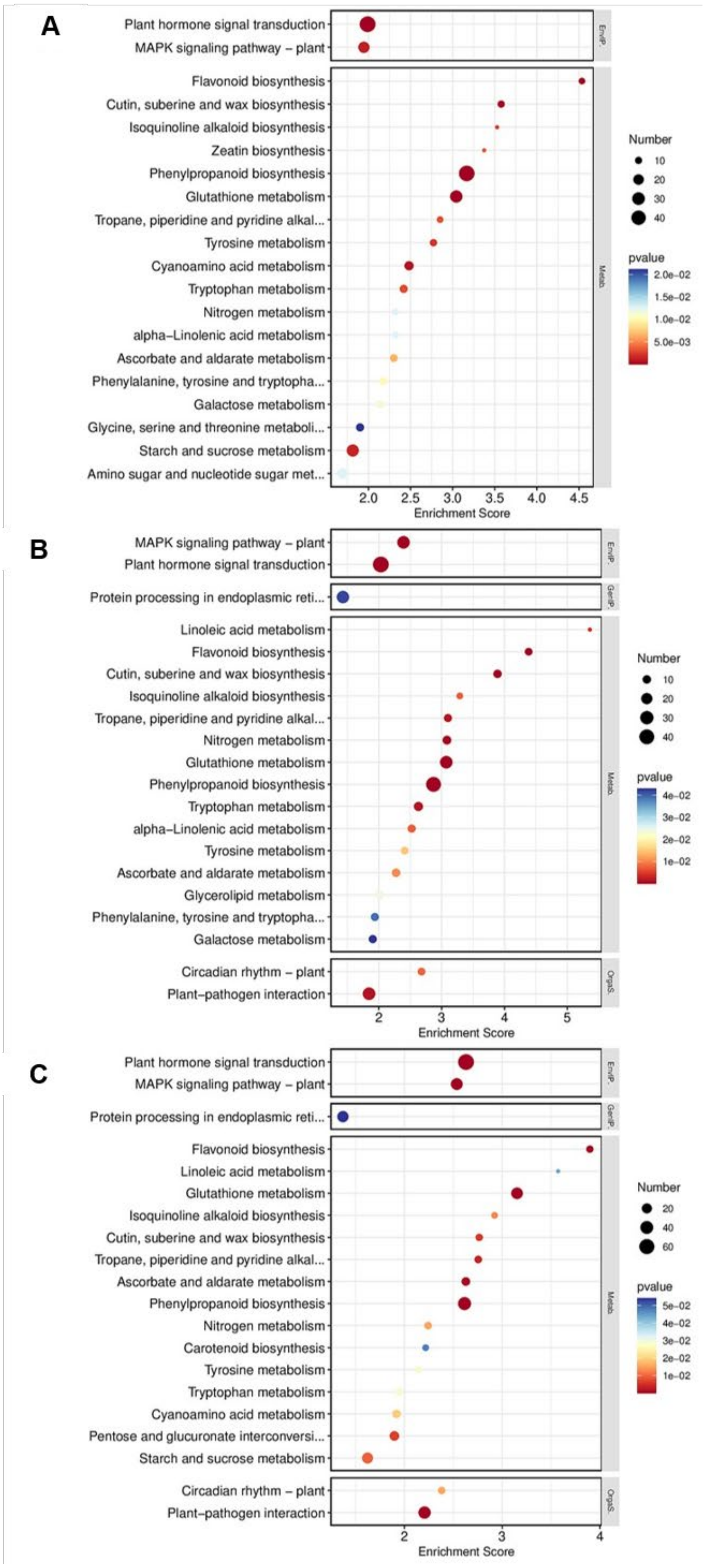
A
Plant hormone signal transduction
MAPK signaling pathway – plant
Flavonoid biosynthesis
Cutin, suberine and wax biosynthesis
Isoquinoline alkaloid biosynthesis
Zeatin biosynthesis
Phenylpropanoid biosynthesis
Glutathione metabolism
Tropane, piperidine and pyridine alkal...
Tyrosine metabolism
Cyanoamino acid metabolism
Tryptophan metabolism
Nitrogen metabolism
alpha–Linolenic acid metabolism
Ascorbate and aldarate metabolism
Phenylalanine, tyrosine and tryptopha...
Galactose metabolism
Glycine, serine and threonine metaboli...
Starch and sucrose metabolism
Amino sugar and nucleotide sugar met...
EnvIP.
Metab.
2.0
2.5
3.0
3.5
4.0
4.5
Enrichment Score
Number
10
20
30
40
pvalue
2.0e-02
1.5e-02
1.0e-02
5.0e-03
B
MAPK signaling pathway – plant
Plant hormone signal transduction
Protein processing in endoplasmic reti...
Linoleic acid metabolism
Flavonoid biosynthesis
Cutin, suberine and wax biosynthesis
Isoquinoline alkaloid biosynthesis
Tropane, piperidine and pyridine alkal...
Nitrogen metabolism
Glutathione metabolism
Phenylpropanoid biosynthesis
Tryptophan metabolism
alpha–Linolenic acid metabolism
Tyrosine metabolism
Ascorbate and aldarate metabolism
Glycerolipid metabolism
Phenylalanine, tyrosine and tryptopha...
Galactose metabolism
Circadian rhythm – plant
Plant–pathogen interaction
EnvIP.
GenIP.
Metab.
OrgaS.
2
3
4
5
Enrichment Score
Number
10
20
30
40
pvalue
4e-02
3e-02
2e-02
1e-02
C
Plant hormone signal transduction
MAPK signaling pathway – plant
Protein processing in endoplasmic reti...
Flavonoid biosynthesis
Linoleic acid metabolism
Glutathione metabolism
Isoquinoline alkaloid biosynthesis
Cutin, suberine and wax biosynthesis
Tropane, piperidine and pyridine alkal...
Ascorbate and aldarate metabolism
Phenylpropanoid biosynthesis
Nitrogen metabolism
Carotenoid biosynthesis
Tyrosine metabolism
Tryptophan metabolism
Cyanoamino acid metabolism
Pentose and glucuronate interconversi...
Starch and sucrose metabolism
Circadian rhythm – plant
Plant–pathogen interaction
EnvIP.
GenIP.
Metab.
OrgaS.
2
3
4
Enrichment Score
Number
20
40
60
pvalue
5e-02
4e-02
3e-02
2e-02
1e-02

Figure S3. KEGG enrichment analysis of DEGs under normal treatment. (A-C) KEGG enrichment analysis of DEGs, identified by the comparison between WT and *gun1* (A), WT and *SALK_047877* (B), WT and *SALK-083115* (C) under normal growth conditions.

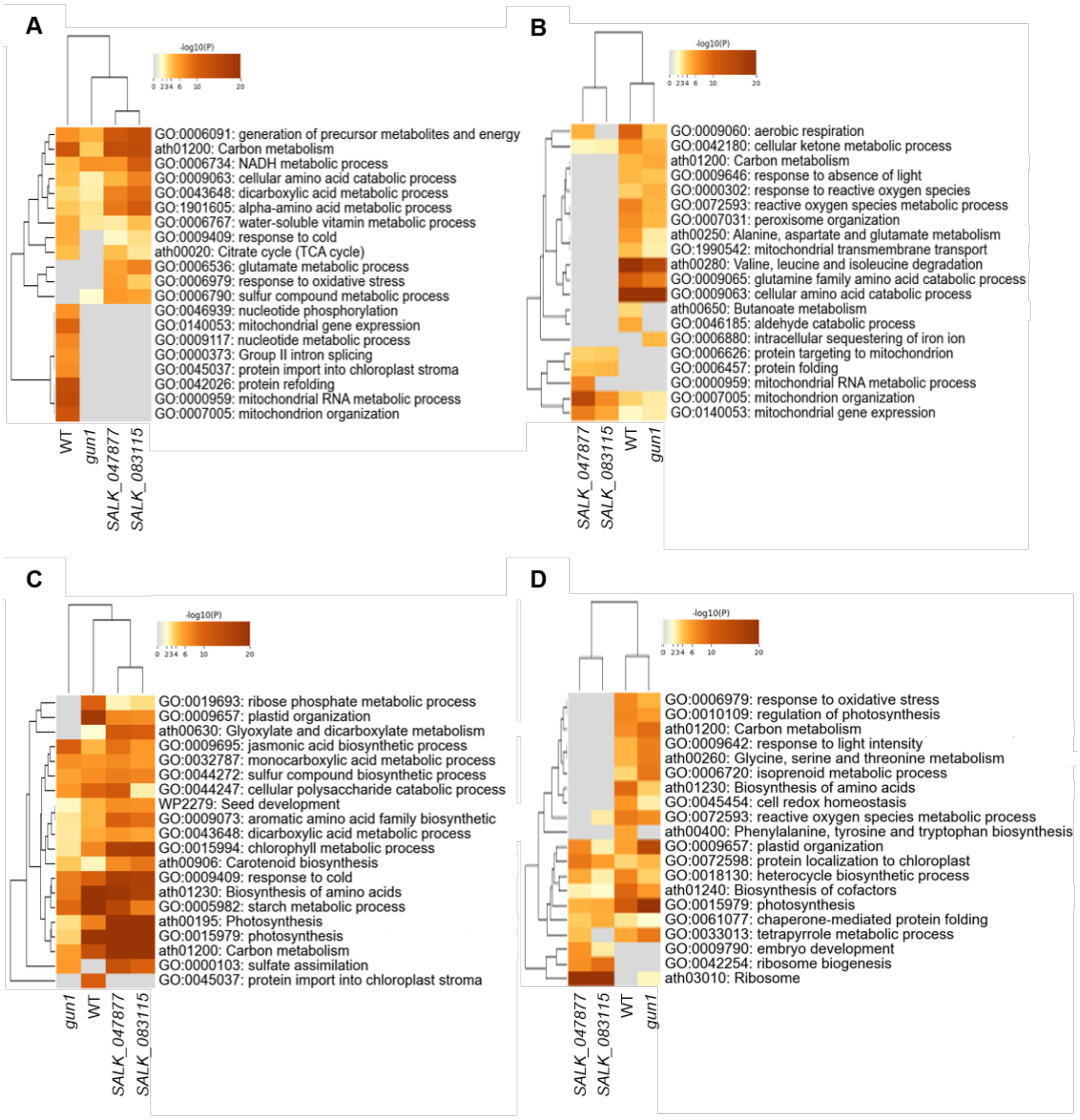

Figure S4. GO enrichment analysis of mitochondria-related and chloroplast-related DEGs in LIN vs control comparison groups. (A) and (B) GO enrichment analysis of down-regulated and up-regulated mitochondria-related DEGs in different comparison groups, respectively. (C) and (D) GO enrichment analysis of down-regulated and up-regulated chloroplast-related DEGs in different comparison groups, respectively.

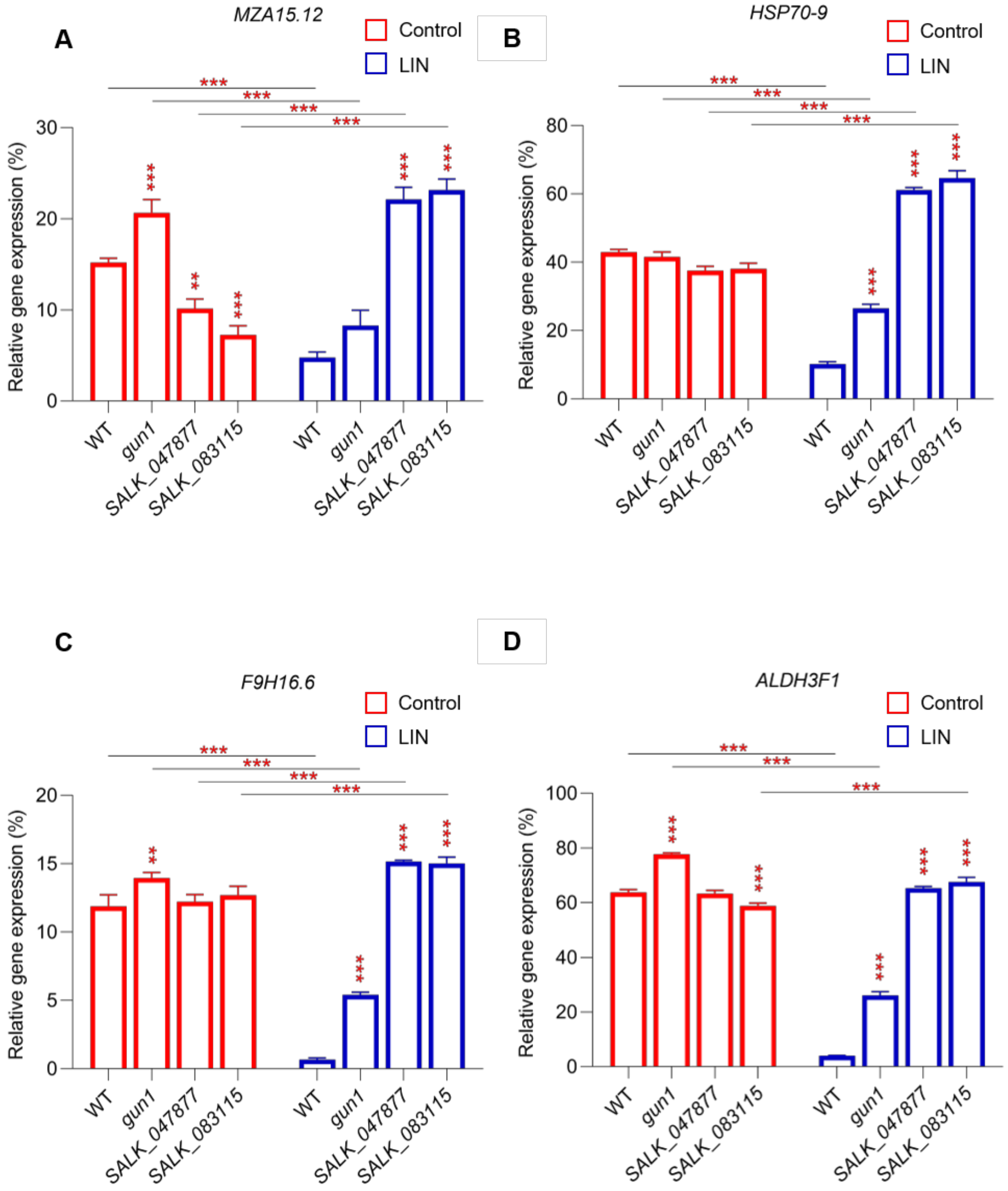

Figure S5. Expression analysis of mitochondria and chloroplast ROS and PAP signaling pathways. **(**A) Analysis of mitochondrial-related genes. (B-D) Analysis of the expression of chloroplast-related and carbon metabolism-related genes. Significant differences are indicated by asterisks (one–way ANOVA with Tukey's multiple comparisons test, * $P< 0.05$, ** $P < 0.01$, and *** $P < 0.001$, n=3).

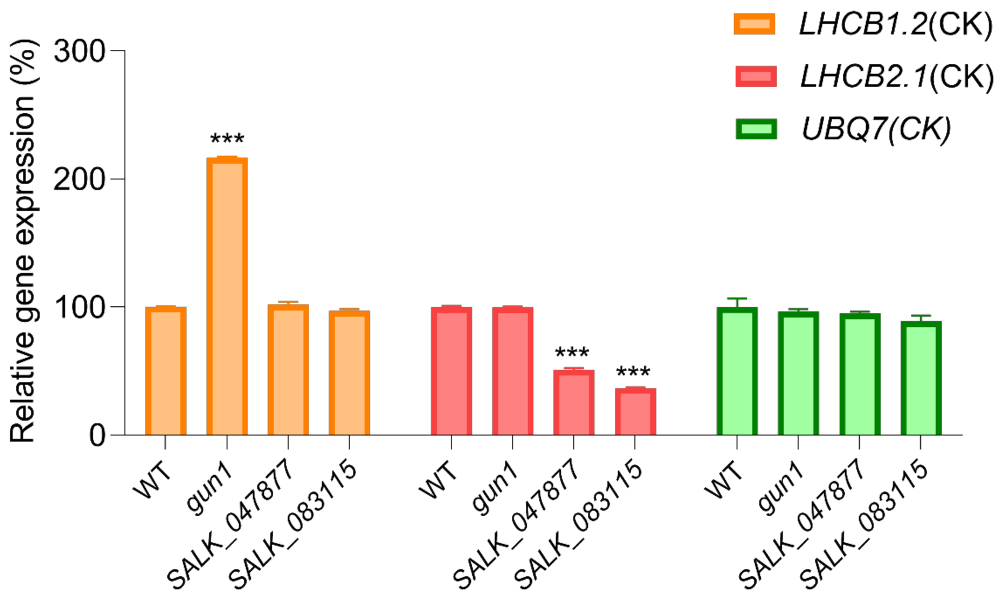

Figure S6. The relative expression level of *LHCB* under control condition. The relative expression level of *LHCB1.2* and *LHCB2.1* in *gun1*, *SALK_047877*, *SALK_083115*, and WT plants under control condition. Significant differences are indicated by asterisks (one–way ANOVA with Tukey's multiple comparisons test, *** $P < 0.001$, n=3).

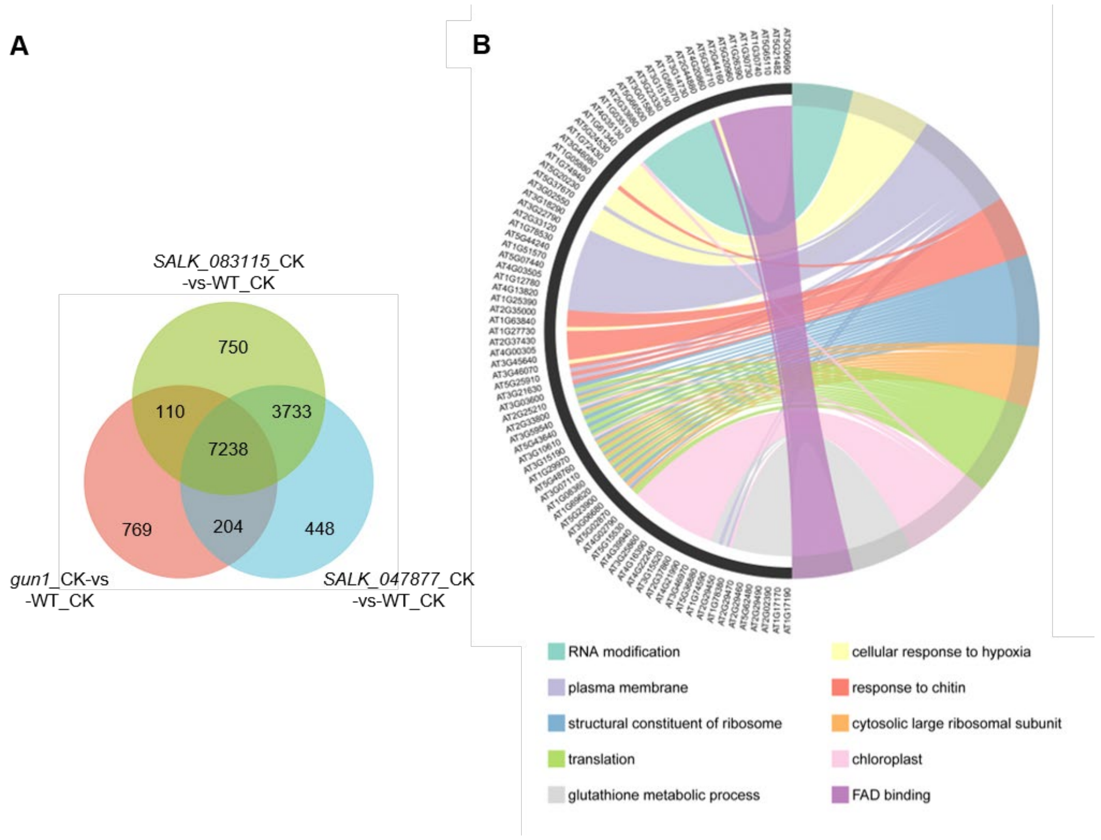

Figure S7. GO enrichment analysis of DEGs, identified by comparison between WT,

gun1, *SALK_047877*, and *SALK-083115* under control condition. (A) Venn diagram analysis of DEGs between WT and mutants under control condition. (B) GO enrichment analysis of significant overlap of DEGs (7238).
